# Breastfeeding counseling and support are associated with continuous exclusive breastfeeding from one week to six months of age among HIV exposed infants in north Gondar zone, Ethiopia: a cross-sectional study

**DOI:** 10.1186/s13006-017-0113-1

**Published:** 2017-04-21

**Authors:** Haregeweyin Genetu, Melaku Kindie Yenit, Amare Tariku

**Affiliations:** 1 Gondar University Hospital, Gondar, Ethiopia; 2 Department of Epidemiology and Biostatistics, College of Medicine and Health Sciences, Institute of Public Health, University of Gondar, Gondar, Ethiopia; 3 Department of Human Nutrition, College of Medicine and Health Sciences, Institute of Public Health, University of Gondar, Gondar, Ethiopia

**Keywords:** Continuous exclusive breastfeeding, Exposed infants, Breastfeeding counseling, Breastfeeding support, Ethiopia

## Abstract

**Background:**

Globally, exclusive breastfeeding prevents 1.3 million child deaths and has an added benefit for people living with the Human Immune Deficiency Virus (HIV) by preventing mother-to-child transmission of HIV. However, literature is scarce in Ethiopia; therefore this study aimed to assess the rate of continuous exclusive breastfeeding from the end of the first week to 6 months of age, among HIV exposed infants (aged 7–20 months) who were followed up in government hospitals of North Gondar Zone, Ethiopia, and associated factors.

**Methods:**

An institutional based cross-sectional study was conducted from February 21 to March 30, 2016. All mother-child pairs (367) attending the chronic HIV care clinic in government hospitals of North Gondar Zone were included in the study. Exclusive breastfeeding was defined as the practice of feeding only breast milk (including expressed breast milk) during the first 6 months and no other liquids and solid foods except medications. However since prelacteal feeding is a deep rooted norm in the study setting, we report continuous exclusive breastfeeding from the end of first week to 6 months of age of infants, ignoring all prelacteal feeding offered at birth. A binary multivariable logistic regression analysis was employed to identify factors associated with exclusive breastfeeding.

**Results:**

The overall prevalence of continuous exclusive breastfeeding among HIV exposed infants was 86.4%. According to the multivariable analysis; breastfeeding counseling (Adjusted Odds Ratio [AOR] = 5.1, 95% Confidence Interval [CI] 1.4, 18.2), breastfeeding support (AOR = 3.7, 95% CI 1.3, 10.5), and not experiencing obstetric problems (AOR = 3.4, 95% CI: 1.3, 8.8) were associated with higher odds of continuous breastfeeding.

**Conclusions:**

In this study, most HIV exposed infants were continuously breastfed from the end of first week to 6 months of age. Breastfeeding counseling, breastfeeding support and experiencing obstetric problems were identified as significant determinants of continuous breastfeeding. Therefore, breastfeeding counseling and support should be strengthened to improve the coverage of optimal exclusive breastfeeding practice. Moreover, prompt diagnosis and treatment of obstetric problems should be initiated.

## Background

Breastfeeding is an essential child survival intervention and provides immunological, psychological, and socio-economic benefits [1–3]. Globally, exclusive breastfeeding prevents 1.3 million child deaths [4]. In the era of the Human Immune Deficiency Virus (HIV) pandemic, breastfeeding is considered as a source of mother-to-child transmission of HIV [1, 5, 6]. As a result, the World Health Organization (WHO) advised mothers infected with HIV to avoid breastfeeding if they were able to afford and safely prepare formula milk [7]. However, mindful of the growing evidence supporting the benefits of EBF, the WHO now recommends HIV positive mothers to exclusively breastfed their infant’s for 6 months, and to take antiretroviral drugs throughout the breastfeeding period and until the child is 12 months of age, to reduce the risk of HIV transmission [6–9].

In spite of this fact, not exclusively breastfeeding continues as a global public health problem, and only 39% of infants are exclusively breastfed [10, 11]. In particular, mixed feeding is still practiced as the norm in developing countries, and it is found to increase the risk of child deaths from under-nutrition, diarrhea, pneumonia and other morbidities [10]. There is a marked regional variation; about 24–80% of infants exposed to HIV are exclusively breastfed in Africa [11–18]. According to the Ethiopian Demographic and Health Survey (EDHS) Report (2011), about 51% of mothers practice EBF [19]. However, some improvements are observed among HIV infected mothers, and the prevalence of EBF is modest in Addis Ababa [20] and West Oromia (68 and 72%, respectively) [21], while it is highest in Gondar (89.5%) [22] and Mekele (90%) [11]. However, the prevalence of exclusive breastfeeding in Southern Ethiopia is even lower than the national average of 48% [7].

Studies have demonstrated that exclusive breastfeeding affected by various factors. The mother’s age [20], knowledge on the benefit of exclusive breastfeeding [12, 23], occupation [7, 12, 20, 22], urban or rural residence [24], lack of education [20], income [20], and the high cost of formula milk [13] are some the sociodemographic factors significantly associated with exclusive breastfeeding. It is also strongly related to the level of maternal healthcare utilization and morbidities, accordingly the mode and place of delivery [18, 20, 22, 25], prenatal [20] and postnatal healthcare utilization [20], receiving infant feeding counseling [20, 25, 26], breastfeeding support [20, 22], facing obstetric complications and other maternal morbidities [20, 25] and history of infant illness in the first 6 months [20, 27] are correlated with EBF. Among the psychosocial determinants, fear of stigma and discrimination [9, 14, 25] and use of herbal medicines [25] are inversely related with exclusive breastfeeding.

In Ethiopia about 1.5% of adults (15–49 years) are HIV positive and the prevalence is higher among women (1.9%) [19]. To reduce the risk of mother-to-child transmission of HIV and AIDS related child mortality, the country designed a guideline which advocates exclusive breastfeeding for the first 6 months, though the practice remains sub-optimal [20–22, 27]. Exclusive breastfeeding among HIV positive mothers is not well investigated; the available studies have been conducted on urban dwellers [20, 22, 27], while poorer child feeding practice, lower literacy rate and maternal health care utilization are documented in the rural inhabitants [19]. Therefore this study aimed to determine the prevalence of continuous exclusive breastfeeding and associated factors from the end of first week to 6 months of age among HIV exposed infants (7–20 months) who had follow up in government hospitals of North Gondar Zone, Ethiopia.

## Methods

### Study setting and design

An institutional based cross-sectional study was conducted from 21 February to 30 March 2016 in the three hospitals of North Gondar Zone, northwest Ethiopia. The zone has 126 health centers and 6 hospitals providing health services to the community. Among the total six hospitals in the study area, only three (Metema Hospital, Dabark Hospital and Gondar University Hospital) are currently providing Anti-Retro Viral Treatment (ART) and Prevention of Mother to Child Transmission (PMTCT) of HIV services. A total of 392 HIV infected mothers with children aged 7–20 months were registered for HIV care and support.

### Sample size and sampling procedure

All HIV positive mothers who had a child aged between 7 and 20 months and attended the chronic HIV care clinic were the population under study. Mother-child pairs were recruited from the three public hospitals providing HIV care in North Gondar Zone. The sample size was calculated using Epi-Info version 3.7 by considering the following assumptions; the prevalence of EBF among HIV exposed infants was 68% [20], 95% level of confidence, 5% margin of error, and 10% non-response rate. Finally, the minimum sample size of 367 was obtained. To employ the systematic random sampling technique, a sampling fraction (K^th^ = N/n) was computed by dividing the total number of mother-child pairs enrolled for HIV care in the three hospitals to the total sample size, and it was 1.07. As a result, all eligible mother-child pairs attending the hospitals during the study period were included consecutively.

### Data collection procedure

A structured interviewer-administered questionnaire was used to collect data. To maintain consistency, the questionnaire was first translated from English to Amharic (the native language of the study area) and was retranslated to English by professional translators and Public Health experts. Six clinical nurses as data collectors and two health officers as supervisors were recruited for the study. Two days intensive training regarding the objective of the study, confidentiality of information, and techniques to conduct interview was given to the data collectors and supervisors.

To address the ethical issues, the data collectors were recruited among the permanent employees of the respective hospitals. The data collectors might be prone to commit social desirability bias, and efforts were made to equip the data collectors with techniques to minimize this risk. Accordingly, all the study participants were informed about the objective of the study. The study was not aimed to evaluate their adherence to counseling and treatment, and their response would not affect their treatment. Moreover, data collectors created a conducive environment by keeping the mothers apart and making them comfortable during data collection. For a few mothers who faced difficulties in remembering the right time of cessation of EBF, data collectors carried out different probing mechanisms to help them to recall, thereby minimizing recall bias. Relating the time of initiation of additional (complementary) food to known public events and immunization schedules were some of the probing techniques used by data collectors.

The questionnaire was also piloted on 5% of the total sample out of the study area and the acceptability and applicability of the procedures and tools were evaluated during pretest.

### Data analysis and study variables

According to the WHO criteria, exclusive breastfeeding is defined as the practice of feeding only breast milk (including expressed breast milk) during the first 6 months and no other liquids and solid foods except medications [28]. Accordingly, we asked mothers the question “When did you first introduce any solid, semi-solid, or fluid (including water) to [child’s name] in addition to breast milk”. However, prelacteal feeding is widely practiced in the study area [19]. Therefore, using a lifelong indicator would underestimate the proportion of infants being exclusively breastfed. We decided to excluded anything given to the infant in the first 2–3 days of life (prelacteal feeding) [29]. Therefore, this study determined the continuous exclusive breastfeeding period from the end of first week to 6 months of age among HIV exposed infants (aged 7–20 months) who had follow up in government hospitals of North Gondar Zone.

Eleven questions were used to determine the mother’s Infant and Young Child Feeding (IYCF) knowledge. Mothers were asked about the modes of mother to child transmission of HIV, benefit of breastfeeding, timely initiation of breastfeeding, colostrum feeding and it’s health benefit, optimal duration of exclusive breastfeeding, timely initiation of complementary feeding, type of food to start complementary feeding, how to feed a child and optimal duration of continued breastfeeding. Using Principal Component Analysis (PCA), the factor scores were summed and ranked to terciles as lowest, medium and highest. Likewise, the household wealth status was computed using a composite indicator for urban and rural residents by considering household assets and size of agricultural land. PCA was performed to define the household wealth status into lowest, middle and highest.

Data were entered into Epi-Info version 3.5.3 and exported to Statistical Package for Social Sciences (SPSS) version 20 for further analysis. Data cleaning was done by running frequencies. Descriptive statistics, including frequencies and proportions were computed to summarize variables. A binary logistic regression model was fitted. Both bivariable and multivariable logistic regression analyses were done, and variables with a *p*-value of < 0.2 in the bivariable analysis were subjected to the multivariable analysis. Both Crude Odds Ratio (COR) and Adjusted Odds Ratio (AOR) with a 95% Confidence Interval (CI) were estimated to show the strength of association. A *p*-value of < 0.05 was used to identify variables that showed statistical significance in the multivariable analysis.

## Results

A total of 367 mother-child pairs were included in the study. The mean (± Standard Deviation, SD) age of the mothers and the children was 31.3 (±4.4) years and 12.9 (±4.1) months, respectively. In this study, about three-fourths of the mothers (74.9, 77.7 and 82.3%, respectively) were married, urban inhabitants, and aged 20–35 years. Nearly half (44.8%) of mothers were outdoor workers (Table 1).

**Table 1:** Sociodemographic and economic characteristics of HIV positive mothers with children aged 7 to 20 months attending public hospitals in North Gondar Zone, Ethiopia, 2016 (*n* = 367)

Variables	Frequency	Percentage
Child sex		
Male	193	52.6
Female	174	47.4
Child age		
7–12 months	185	50.4
13–20 months	182	49.6
Mean (± SD) age 12.9(±4.1) months		
Maternal age		
20–35 years	302	82.3
36–43 years	65	17.7
Mean ± SD for mothers was 31.3(±4.4) years		
Number of parity		
1–3	325	88.6
4–6	42	11.4
Number of under five children		
1	242	65.9
>1	125	34.1
Family size		
≤5	269	73.3
>5	98	26.7
Residence		
Urban	285	77.7
Rural	82	22.3
Marital status		
Currently married	275	74.9
Currently unmarried^c^	92	25.1
Religion		
Orthodox	296	80.7
Muslim	52	14.2
Protestant	19	5.1
Ethnicity		
Amhara	286	77.9
Kemant	62	16.9
Tigre	12	3.3
Oromo	7	1.9
Wealth status		
Poor	122	33.3
Medium	130	35.4
Rich	115	31.3
Maternal education		
Illiterate	110	30
Literate	257	70
Paternal education		
Illiterate	48	13.1
Literate	319	86.9
Maternal employment		
Employee^b^	96	26.2
Housewife	166	45.2
Outdoor workers^a^	105	28.6
Paternal employment		
Employee^b^	149	40.6
Outdoor workers^a^	218	59.4

^a^Merchants and daily laborers

^b^Governmental and non-governmental employees

^c^single, divorced, and widowed

A substantial proportion (96.5%) of mothers had at least one antenatal care visit, of which 94.6% were counseled about EBF. Almost all (97.3%) of respondents gave birth at healthcare institutions; and cesarean-section delivery was reported by 21.5%. About 47.4% of mothers had an obstetric problem (Table 2). Despite efforts to prevent mother-to-child transmission of HIV, about 1.9% of exposed infants were HIV positive. All (100%) of the HIV infected children were from mothers who did not receive any breastfeeding support and gave birth virginally. A higher proportion of HIV infection was noted among children who were given prelacteal feeds, whose mothers were illiterate (71.4%) and who experienced an obstetric problem (57.1%) (Table 3).

**Table 2:** Healthcare utilization and morbidity related characteristics of HIV positive mothers with children aged 7 to 20 months attending public hospitals in North Gondar Zone, Ethiopia, 2016 (*n* = 367)

Variables	Frequency	Percentage
Antenatal care visit		
Yes	354	96.5
No	13	3.5
Number of antenatal care visits		
1–3	72	20.3
≥4	282	79.7
Counseling about infant feeding		
Yes	317	89.5
No	37	10.5
Counseling about exclusive breastfeeding		
Yes	347	94.6
No	20	5.4
Place of delivery		
Health institution	357	97.3
Home	10	2.7
Gestational age		
Preterm	1	0.2
Term	357	97.3
Post term	9	2.5
Mode of delivery		
Spontaneous vaginal	174	47.4
Assisted vaginal delivery	114	31.1
Cesarean section	79	21.5
History of obstetrics problem		
Yes	174	47.4
No	193	52.6
Type of obstetric problem		
Prolonged labor	138	79.3
Ante partum hemorrhage	14	8.0
Postpartum hemorrhage	20	11.6
Others^a^	2	1.1
Breast problem		
Yes	45	12.3
No	322	87.7
Child weight		
2.5–3.0 kg	222	60.5
>3.0 kg	110	30.0
Not recorded	35	9.5
Disclosing HIV status		
Yes	309	84.2
No	58	15.8
For whom HIV status disclosed^b^		
Spouse	261	71.1
Family	219	59.6
Community	16	4.4
Friend	53	14.4
Others	32	8.4
Postnatal care		
Yes	266	72.5
No	101	27.5
History of infanthood morbidity		
Yes	120	32.7
No	247	67.3
Healthcare access		
≤2 h	233	63.5
>2 h	134	36.5
Breastfeeding support received		
Yes	317	90.6
No	33	9.4
Source of breastfeeding support		
Spouse	251	68.4
Family	286	78.8
Community^c^	1	0.3
Friends	85	23.2
Health professionals	222	60.5
Mothers decide to breastfeed		
Yes	325	88.6
No	42	11.4

^a^Others-pregnancy induced hypertension and acute febrile illness

^b^disclose their HIV status for more than one person

^c^Support from different people

**Table 3:** Distribution of child HIV status by the selected maternal characteristics in North Gondar Zone public hospitals, Ethiopia

Variables	HIV status of the child	
	Positive # (%)	Negative # (%)
Prelacteal feeding		
Yes	4 (57.1)	66 (18.3)
No	3 (42.9)	294 (81.7)
Mother’s education		
Illiterate	5 (71.4)	105 (29.2)
Literate	2 (28.6)	255 (70.8)
Counseling about exclusive breastfeeding		
Yes	0 (0)	347 (96.4)
No	7 (100.0)	13 (3.6)
Mode of delivery		
Vaginal delivery	7 (100.0)	167 (46.4)
Others^a^	0 (0)	193 (43.6)
History of obstetric problem		
Yes	4 (57.1)	170 (47.2)
No	3 (42.9)	190 (42.8)

^a^Cesarean section and instrumental delivery

The study demonstrated that, about 86.4% (95% CI: 82.9, 89.9%) of HIV exposed infants were continuously exclusively breastfed from the end of first week to 6 months of age. Two-thirds (72.8%) of mothers initiated breastfeeding within an hour of delivery, and about 79.6% of infants were given colostrum. However, prelacteal feeding was given to 19.1% of infants (Table 4).

**Table 4:** Breastfeeding practice of children aged 7 to 20 months attending public hospitals in North Gondar Zone, Ethiopia, 2016 (*n* = 367)

Variables	Frequency	Percentage
Continuous exclusive breastfeeding		
Yes	317	86.4
No	50	13.6
Ever breastfed		
Yes	352	95.9
No	15	4.1
Reasons for not breastfeeding (*n* = 15)		
Shortage of breast milk	2	13.3
Mother was busy	7	46.7
Not willing to breastfeed	2	13.3
Health problem of the mother	3	20
Health problem of the child	1	6.7
Colostrum given		
Yes	292	79.6
No	75	20.4
Prelacteal feeding		
Yes	70	19.1
No	297	80.9
Type of prelacteal food given^a^		
Butter	8	11.4
Plain water	25	35.7
Sugar with water	40	57.1
Abesh^b^	11	15.7
Traditional medicine	7	10
Frequency of breastfeeding per day		
≥8 times	317	90.1
<8 times	35	9.9
Initiation of breastfeeding		
Within 1 h	254	72.8
After 1 h	95	27.2
Mothers IYCF knowledge		
Good	136	37
Medium	132	36
Poor	99	27

^a^Multiple responses

^b^Abesh is made from a roosted fenugreek which is powdered, fermented, and diluted with water

Both bivariable and multivariable analyses were done to identify factors associated with continuous exclusive breastfeeding. Accordingly, the result of bivariable analysis noted that counseling about exclusive breastfeeding, colostrum feeding, experiencing an obstetric problem, decision to breastfeed, breastfeeding support, place of delivery, and infant illness were significantly associated with the practice of continuous exclusive breastfeeding. However, only breastfeeding support, breastfeeding counseling, and experiencing an obstetric problem remained significantly and independently associated with exclusive breastfeeding in the multivariable analysis. Consequently, the odds of continuous EBF were higher among HIV infected mothers who received prenatal EBF counseling (AOR = 5.1; 95% CI: 1.4, 18.2) compared to those who did not receive counseling. Similarly, obtaining breastfeeding support (AOR = 3.7, 95% CI: 1.3, 10.5) and not experiencing an obstetric problem (AOR = 3.4, 95% CI: 1.3, 8.8) were associated with increased odds of mothers with a continuous exclusive breastfeeding practice (Table 5).

**Table 5:** Factors associated with continuous EBF among HIV positive mothers with children aged 7 to 20 months attending public hospitals in North Gondar Zone, Ethiopia, 2016 (*n* = 367)

Variables	EBF^a^		COR^b^ (95% CI)	AOR^c^ (95% CI)
	Yes	No		
Counseling about EBF				
Yes	304	43	3.8 (1.4,10.1)	5.1 (1.4, 18.2)**
No	13	7	1	1
History of obstetric problem				
Yes	137	37	1	1
No	180	13	3.7 (1.9,7.3)	3.4 (1.3, 8.9)**
Decision to BF				
Yes	290	35	4.6 (2.2,9.5)	1.6 (0.5,5.2)
No	27	15	1	1
Support to BF				
Yes	288	30	6.6 (3.3,13.1)	3.7 (1.3,10.5)**
No	29	21	1	1
History of infanthood illness				
Yes	100	32	1	1
No	217	18	3.9 (2.1,7.2)	1.45 (0.6,3.6)
Colostrum given				
Yes	262	30	3.5 (1.8,6.5)	1.92 (0.7,5.3)
No	55	20	1	1
Place of delivery				
Health institution	312	45	1	1
home	5	5	0.14 (0.1,0.5)	0.7 (0.1,6.9)

^a^Exclusive breastfeeding

^b^Crude Odds Ratio

^c^Adjusted Odds Ratio

**significant at a *p*-value of < 0.05

## Discussion

Exclusive breastfeeding has been considered as one of the proven child survival interventions [10, 30]. Ethiopia has adopted these established facts and set a target to improve EBF to 70% by 2020 [31]. This study showed that, the prevalence of continuous EBF among infants exposed to HIV was 86.4% which was higher than the national target. The finding was also consistent with other reports from Ethiopia: Gondar (89.5%) [22] and Mekele (90%) [31]. This might be related to the contextual similarities in maternal healthcare utilization among study settings.

However, the prevalence of continuous EBF was higher than what was reported from Addis Ababa (68%) [20], Southern Ethiopia (48%) and West Oromia (72%) [21]. The observed discrepancy might be attributed to efforts made by the government in promoting the optimal child feeding practice for HIV infected mothers mainly in the PMTCT, exposed infant, and ART clinics [32, 33]. Likewise, the finding was higher than the reports of other developing countries, such as India (44%) [34], Tanzania (24–46%) [16], Kenya (80.4%) [15], Ghana (60%) [13] and Nigeria (61%) [14]. This is probably due to the fact that, there is higher cultural acceptability of breastfeeding in this study area. Furthermore, the difference in EBF practice could be related to the variations in the study design. The later studies employed a cohort study design and ascertained exclusive breastfeeding practice prospectively [15, 34], while this study measured EBF retrospectively using a cross-sectional study design. Therefore, the increased prevalence of EBF in the current study could be related to the retrospective ascertainment of the EBF practice [29].

The result of the adjusted analysis showed that, the odds of continuous exclusive breastfeeding were higher among mothers who received prenatal EBF counseling. The finding was supported by reports elsewhere [14, 17, 26, 35, 36]. In fact, birth preparedness and breastfeeding counseling are important components of prenatal care, and the repeated visits also help to bring the intended maternal behavioral change towards the optimal breastfeeding practice. The impact of prenatal care mainly operates through the role of clinicians in providing the correct and up-to-dated IYCF information to the mothers [35, 37]. In Ethiopia, implementation of the urban Health Extension Program steps-up utilization of antenatal care, which creates an extra benefit in bringing the promotive health services, including breastfeeding counseling to the household level [30, 38].

Similarly, the higher odds of continuous exclusive breastfeeding were reported among mothers who had no history of obstetric problems. The finding was consistent with the report from Addis Ababa [20]. The possible explanation might be attributed to the psychological effects of obstetric problems, such as depression, lack of confidence or worry leading to delayed initiation of breastfeeding. In fact, the delayed initiation of breastfeeding was found to negatively affect the duration of EBF [39]. Moreover, when mothers encountered obstetrics problems, they commonly perceived that their infants are not getting enough milk, and therefore they may fail to provide breast milk.

This study also affirmed that continuous exclusive breastfeeding rate was higher among mothers who received breastfeeding support. This finding was comparable with study conducted in Malawi [25] and research done in southern Ethiopia [24]. A similar finding was also reported from Swaziland [36], in which social support increased the ease of breastfeeding. In fact, socio-cultural and economical support has a great role in obtaining the full benefits of breastfeeding, and the profound effect in maintaining optimal breastfeeding was noted in case of family support [35, 37].

Of the commonly documented determinants of EBF, maternal health care utilizations, such as antenatal care and institutional delivery were consistently reported by various studies [18, 20, 22, 25, 40]. But, these variables did not show significant association with continuous EBF in the current study. The discrepancy might be attributed to difference in the maternal health care utilization, in which almost all of the mothers had antenatal and postnatal follow up, and gave birth in a healthcare facility. However in most of the above study settings, utilization of maternal health services is sub-optimal.

The study tried to show the exclusive breastfeeding practice in a wider study area, particularly including the rural residents. However, it was not free from some of the limitations. Consequently, recall bias and social desirability bias are the possible limitation of the study.

## Conclusions

In this study, a high proportion of HIV exposed infants were exclusively breastfed from the end of first week to 6 months of age. Breastfeeding counseling, breastfeeding support and experiencing an obstetric problem were the key factors associated with continuous exclusive breastfeeding. Therefore, intensifying breastfeeding counseling and support is essential to improve exclusive breastfeeding practices. Moreover, prompt diagnosis and treatment of obstetric problems should be encouraged.

## Abbreviations

AIDS: Acquired immune deficiency syndrome
ANC: Antenatal care
AOR: Adjusted odds ratio
ART: Antiretroviral treatment
CI: Confidence interval
HIV: Human immune deficiency virus
IYCF: Infant young child feeding
PMTCT: Prevention of Mother To Child Transmission
SPSS: Statistical package for social sciences
WHO: World Health Organization
